# Interpreting Blood Culture Results as Early Guidance for Infective Endocarditis

**DOI:** 10.1001/jamanetworkopen.2025.8079

**Published:** 2025-05-01

**Authors:** Sarah R. Freling, Iman Richie, Daniel Norwitz, Catherine P. Canamar, Josh Banerjee, Kusha Davar, Devin Clark, Brad Spellberg

**Affiliations:** 1 Department of Infectious Diseases, Los Angeles General Medical Center, Los Angeles, California; 2 Department of Medicine, Keck School of Medicine of USC, Los Angeles, California; 3 Department of Population Public Health Sciences, Keck School of Medicine of USC, Los Angeles, California

## Abstract

**Importance:**

Few bedside tools with defined accuracy have been described that are useful to alter bayesian prior probability for infective endocarditis (IE) in patients with bacteremia.

**Objective:**

To evaluate the accuracy of simple blood culture parameters to guide pretest probability of IE.

**Design, Setting, and Participants:**

This multicenter, retrospective case-control study of blood culture data in adults with IE vs without IE was conducted at 3 acute care public hospitals in the Los Angeles County Department of Health Services between December 2018 and August 2022. Patients were individuals aged 18 years or older who had positive blood cultures and met the inclusion criteria, including cases who met the Duke criteria for definite or possible IE, and control cases who did not have concern for endocarditis.

**Exposures:**

Positive blood cultures for methicillin-susceptible *Staphylococcus aureus*, methicillin-resistant *S aureus*, *Enterococcus faecalis*, low-risk *Streptococcus* species, or high-risk *Streptococcus* species.

**Main Outcomes and Measures:**

The primary outcome was the negative likelihood ratio (LR) of having endocarditis based on the number of positive blood cultures on admission. Positive LRs, evaluation of 2 or more of 4 bottles positive on admission, bacteremia lasting for at least 2 days, and combination groups were secondary outcomes.

**Results:**

A total of 252 eligible patients with IE (182 male [72%]; median [IQR] age, 54 [38-65] years), including 164 definite and 88 possible IE cases, and 455 controls (321 male [71%]; median [IQR] age, 53 [41-63] years) were identified. The negative LR point estimates for having IE with only 1 of 4 positive blood culture bottles on admission ranged from 0.05 (95% CI, 0.01-0.37) for *E faecalis *to 0.12 (95% CI, 0.03-0.49) for methicillin-susceptible *S aureus*. Sensitivity analysis of cases restricted to definite IE found similar results. Blood culture clearance by day 2 also had modestly helpful negative LRs for methicillin-resistant *S aureus *(0.24; 95% CI, 0.13-0.42) and *Enterococcus* species (0.34; 95% CI, 0.21-0.56). If patients had 4 of 4 bottles positive on admission, positive LRs were helpful for *Enterococcus* species (LR, 4.21; 95% CI, 2.53-7.02) and high-risk streptococci (LR, 5.35; 95% CI, 3.39-8.42) and for all organisms with persistent bacteremia (with LRs ranging from 1.78 [95% CI, 1.36-2.34] to 9.60 [95% CI, 3.43-44.60]). If both were true, the positive LRs ranged from 1.63 (95% CI, 1.17-2.28) to 8.59 (95% CI, 3.43-21.55) for all organisms.

**Conclusions and Relevance:**

In this case-control study of patients with and without IE, the number of initial positive blood culture bottles and days to culture clearance were helpful to adjust pretest probability of IE. These findings may help guide diagnostic and therapeutic decisions around bacteremia early during hospitalization.

## Key Points

**Question:**

Can the number of initial positive blood cultures and the number of days until culture clearance help guide pretest probability for infective endocarditis?

**Findings:**

In this multicenter case-control study of 707 adults (252 with and 455 without infective endocarditis), if only 1 of 4 blood cultures was positive on admission or if bacteremia was not persistent, for most organisms the negative likelihood ratio for infective endocarditis was very low.

**Meaning:**

These findings suggest that in an era of diagnostic and antimicrobial stewardship, these simple blood culture results, which are readily available at the bedside in real time, can help guide diagnostic and therapeutic decisions around bacteremia early during hospitalization.

## Teaser

This case-control study examines the accuracy of simple blood culture parameters to guide pretest probability of infective endocarditis.

## Introduction

Determining which patients with bacteremia require additional evaluation for infective endocarditis (IE) is a complex task.^1,2,3^ The limited observational data available do not provide information on the diagnostic accuracy (ie, sensitivity, specificity, and positive and negative likelihood ratios [LRs]) of blood culture information differentiated by pathogen.^4,5,6,7,8,9^ One recent cohort study^10^ and one meta-analysis^11^ reported the association of persistently positive blood cultures with risk of IE. However, neither study evaluated the number of blood culture bottles initially positive and did not differentiate culture results by pathogen. Only one study^8^ was found specifically evaluating low-grade bacteremia, which showed decreased risk of IE with 1 of 4 *Staphylococcus aureus* bacteremia, but did not evaluate additional organisms or provide LRs to guide pretest probabilities.

Published predictive scores for IE use complex algorithms and point systems that are difficult to implement at the bedside.^12,13,14,15,16,17,18,19^ We sought to evaluate the diagnostic accuracy of several simple parameters of blood culture results, stratified by pathogen, to help clinicians adjust bayesian prior probability estimates to inform diagnostic and therapeutic considerations for risk of IE.

## Methods

### Study Design

For this case-control study, we conducted a retrospective record review of Cerner-based electronic medical records for patients with bacteremia cared for at 3 acute care safety-net hospitals: Los Angeles General Medical Center, Harbor–University of California Los Angeles Medical Center, and Olive View–University of California Los Angeles Medical Center (all in Los Angeles, California). The University of Southern California institutional review board approved this study as expedited and with a waiver of informed consent because the data were deidentified, in accordance with 45 CFR §46. Our study aimed to follow the Strengthening the Reporting of Observational Studies in Epidemiology (STROBE) reporting guidelines.^20^

To identify patients for inclusion or exclusion, we generated an electronic medical record query for all inpatients with blood cultures drawn from December 2018 to August 2022 that grew typical IE pathogens (eg, *Staphylococcus* species, *Streptococcus* species, and *Enterococcus* species). The resultant query identified 3968 patients with bacteremia, including IE cases that were previously described in a published study^21^ comparing oral vs intravenous-only antibiotic therapy for IE. We randomly selected 482 control patients, deemed not to have IE, from the remaining cases.^21^ Age, sex, race, ethnicity, intracardiac devices, and injection drug use information were obtained for descriptive purposes. Ethnicity and race options were preset by the electronic medical record and were chosen by the patient.

### Inclusion and Exclusion Criteria and Definitions

Case patients with definite IE met 2 major criteria, or 1 major and 3 minor criteria, or 5 minor criteria from the modified Duke criteria published in 2000; possible IE was defined as 1 major criteria and 1 minor criteria, or 3 minor criteria.^22,23,24^ Of note, the 2023 modified Duke criteria were not published before our original study, which identified our cases.^25^ To identify specific controls from among the patients with bacteremia, we conducted medical record reviews to confirm they did not receive diagnoses of definite or possible IE.

For both the IE group and the control group, the organisms of interest were methicillin-susceptible *S aureus* (MSSA), methicillin-resistant *S aureus* (MRSA), *Enterococcus faecalis*, low-risk *Streptococcus* species, and high-risk *Streptococcus* species. According to available literature of the likelihood of IE for specific streptococci, the low-risk *Streptococcus* species included *S pneumoniae, S pyogenes* (group A *Streptococcus*), *S dysgalactiae* (group C *Streptococcus*), *S agalactiae* (group B *Streptococcus*),* S viridans* group (*S salivarius, S anginosus/constellatus, *and *S thermophilus*). The high-risk *Streptococcus* species included *S bovis* (*S gallolyticus *and *S infantarius* [group D *Streptococcus*]), *S viridans* group (*S mitis/cristatus, S sanguinis, S gordonii, S parasanguinis, S sanguinis, *and *S mutans*), *Abiotrophia defectiva*, and *Granulicatella adiacens*.^19,26,27,28^ Of note, if the *S viridans* group was not further delineated, it was placed in the high-risk *Streptococcus* species category.

**Figure 1.**
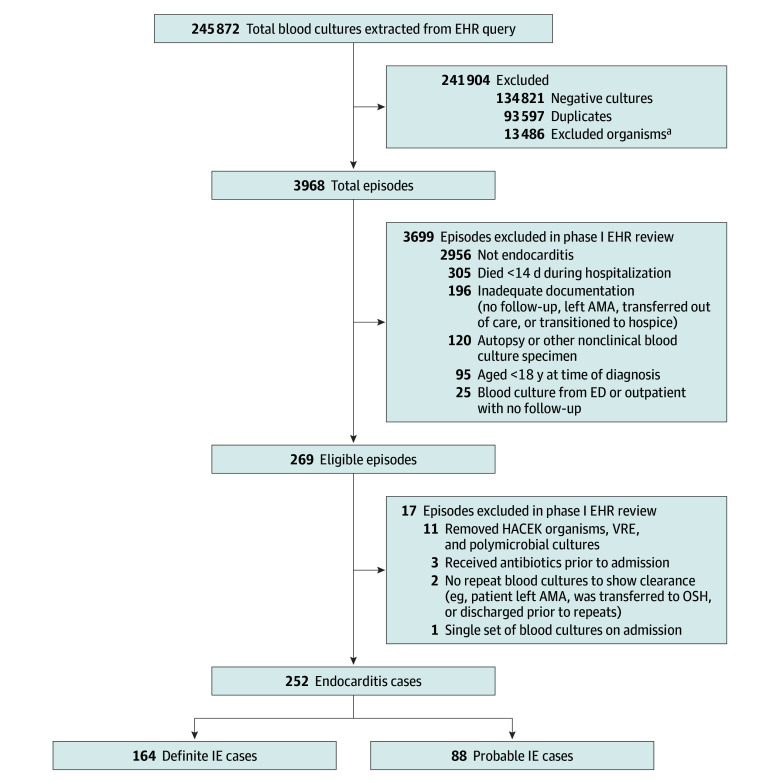
Selection Flowchart for Patients With Infective Endocarditis (IE) AMA indicates against medical advice; ED, emergency department; EHR, electronic health record; HACEK, *Haemophilus* species, *Aggregatibacter* species, *Cardiobacterium hominis*, *Eikenella corrodens*, and *Kingella* species; OSH, outside hospital; VRE, vancomycin-resistant *Enterococcus*. ^a^Includes *Staphylococcus* species, *Streptococcus* species, *Enterococcus* species, and HACEK organisms.

**Figure 2.**
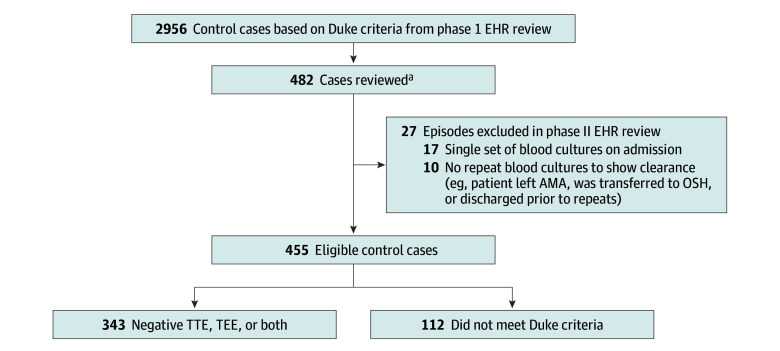
Selection Flowchart for Patients Without Infective Endocarditis AMA indicates against medical advice; EHR, electronic health record; OSH, outside hospital; TEE, transesophageal echocardiogram; TTE, transthoracic echocardiogram. ^a^Includes *Staphylococcus* species, low-risk *Streptococcus* species (ie, *S pneumoniae*, *S pyogenes* [group A *Streptococcus*], *S dysgalactiae* [group C *Streptococcus*], *S agalactiae* [group B *Streptococcus*], and *S viridans* group [*S salivarius*, *S anginosus/constellatus*, and *S thermophilus*]), high-risk *Streptococcus* species (ie, *S bovis* [*S gallolyticus* and *S infantarius*; group D *Streptococcus*], *S viridans *group [*S mitis/cristatus*, *S sanguinis*, *S gordonii*, *S parasanguinis*, *S sanguinis*, and *S mutans*], and nutritionally variant *Streptococcus* species [*Abiotrophia defectiva* and *Granulicatella adiacens*]), and *Enterococcus* species. Ninety to 110 samples of each organism or group were selected to further review.

Exclusion criteria for both cases and controls included postmortem positive blood cultures, age less than 18 years at the time of diagnosis, organisms not included in this study, a single set of blood cultures obtained on admission, no repeat blood cultures obtained (to demonstrate clearance), left against medical advice, transferred out of network, transitioned to hospice, or died early during the hospitalization before sufficient diagnostic evaluation to enable assessment for IE (Figure 1 and Figure 2). However, if IE was confirmed before transfer or transition to hospice, and blood culture clearance was established, those cases were included. Moreover, if a control patient had a clear alternative diagnosis for the bacteremia and there was low clinical suspicion for IE, they were included even without an echocardiogram.

We defined blood cultures on admission as the first 2 sets of blood cultures (4 total bottles) obtained when the patient presented to the hospital. Each blood culture set contained 1 aerobic bottle and 1 anaerobic bottle. We defined culture clearance by day 2 as having at least 1 blood culture bottle positive on day 1, but all blood culture bottles obtained on day 2 resulting in no growth; persistent bacteremia was defined as blood cultures positive for 2 days or more, when indicated. If no blood cultures were obtained on a subsequent day, then that day was counted toward days of bacteremia until culture clearance was confirmed.

### Outcome Measures

The primary outcome was the negative LR (defined as sensitivity divided by 1 minus specificity) of 1 of 4 blood culture bottles positive on admission, stratified by pathogen, for estimating IE.^29^ Prioritized secondary outcome measures included the negative LR and positive LR (defined as 1 minus sensitivity divided by specificity) of other blood culture results, stratified by pathogen. We conducted additional sensitivity analysis focusing only on the cohort of definite IE, excluding possible IE, as well as an analysis focusing on the cohort of controls with negative echocardiogram findings, excluding patients who did not have one. Finally, we evaluated combination criteria, including 1 of 4 positive blood cultures and culture clearance by day 2, compared with all others; 2 of 4 bottles positive and culture clearance by day 2 compared with more than 2 of 4 positive bottles or persistence of bacteremia; and finally, 4 of 4 positive bottles and persistent bacteremia, compared with all others.

### Statistical Analysis

We examined cohort differences on categorical variables using χ^2^ or Fisher exact tests using KyPlot statistical software version 6.0 (KyensLab). Statistical significance was set at α = .05.

## Results

We identified 252 eligible IE episodes (182 male patients [72%]; median [IQR] age, 54 [38-65] years), including 164 definite and 88 possible IE cases (Figure 1, Table 1, and Table 2). These cases were compared with 455 control patients (321 male patients [71%]; median [IQR] age, 53 [41-63] years) without clinical concern for IE, 344 (76%) of whom had negative findings on echocardiogram, and 111 (24%) of whom had such low suspicion for endocarditis that an echocardiogram was not ordered (Figure 2, Table 1, Table 2, and eTable 1 in Supplement 1). The IE and control groups were similar in terms of age, gender, race, and ethnicity (Table 1). There were more patients who had prosthetic valves and injection drug use in the IE cohort (Table 1). In the control group, 30 patients had bacteremia for more than 3 days. These patients were infected with MSSA, MRSA, or *E faecalis*; none was infected with *Streptococcus *species. Alternative diagnoses varied by pathogen (eTable 2 in Supplement 1).

**Table 1.** Demographic Characteristics of Participants

Characteristic	Participants, No. (%)		*P* value
	Infective endocarditis cases (n = 252)	Control cases (n = 455)	
Age, median (IQR), y	54 (38-65)	53 (41-63)	.85
Sex			
Male	182 (72)	321 (71)	.70
Female	70 (28)	134 (29)	
Race and ethnicity			
African American or Black	17 (7)	37 (8)	.64
Asian	13 (5)	20 (4)	
Hispanic or Latin American	159 (63)	264 (58)	
White	12 (5)	28 (6)	
Other, not specified	51 (20)	106 (23)	
Intracardiac prosthesis present on admission			
Prosthetic valve	35 (140)	3 (1)	<.001
Permanent pacemaker or implantable cardiac defibrillator	21 (8)	11 (2)	.06
Injection drug use	55 (22)	38 (8)	<.001

**Table 2.** Case Breakdown

Variable	Cases, No. (%)			
	Total IE cases (n = 252)	Definite IE cases (n = 164)	Possible IE cases (n = 88)	Control cases (n = 455)
Methicillin-susceptible *Staphylococcus aureus*	81 (32)	48 (29)	33 (38)	90 (20)
No. of bottles of 4 positive on admission				
1	2 (1)	1 (1)	1 (1)	19 (4)
2	6 (2)	3 (2)	3 (3)	8 (2)
3	9 (4)	5 (3)	4 (5)	10 (2)
4	64 (25)	39 (24)	25 (28)	53 (12)
Culture clearance by day 2^a^	20 (8)	15 (9)	5 (6)	52 (11)
Persistent bacteremia^b^	14 (6)	7 (4)	7 (8)	13 (3)
Persistent bacteremia ≥3 d	47 (19)	26 (10)	21 (24)	25 (5])
Methicillin-resistant *Staphylococcus aureus*	62 (25)	46 (28)	16 (18)	85 (19)
No. of bottles of 4 positive on admission				
1	2 (1)	0	2 (2)	28 (6)
2	16 (6)	13 (5)	3 (3)	19 (4)
3	4 (2)	3 (2)	1 (1)	9 (2)
4	40 (16)	30 (18)	10 (11)	29 (6)
Culture clearance by day 2	10 (4)	6 (4)	4 (5)	58 (13)
Persistent bacteremia	2 (1)	1 (1)	1 (1)	5 (1)
Persistent bacteremia ≥3 d	50 (20)	39 (24)	11 (13)	22 (5)
*Enterococcus faecalis*	36 (14)	28 (17)	8 (9)	91 (20)
No. of bottles of 4 positive on admission				
1	1 (0.4)	1 (1)	0	48 (11)
2	8 (3)	6 (4)	2 (2)	16 (4)
3	2 (1)	0	2 (2)	12 (3)
4	25 (10)	21 (13)	4 (5)	15 (3)
Culture clearance by day 2	11 (4)	6 (4)	5 (6)	81 (18)
Persistent bacteremia	7 (3)	5 (3)	2 (2)	5 (1)
Persistent bacteremia ≥3 d	18 (7)	17 (10)	1 (1)	5 (1)
Total *Streptococcus* species	73 (29)	43 (26)	30 (34)	189 (42)
Low-risk *Streptococcus *species^c^	25 (10)	14 (9)	11 (13)	80 (18)
No. of bottles of 4 positive on admission				
1	1 (0.4)	0	1 (1)	27 (6)
2	2 (1)	0	2 (2)	10 (2)
3	1 (0.4)	0	1 (1)	10 (2)
4	21 (8)	14 (9	7 (8)	33 (7)
Culture clearance by day 2	19 (8)	12 (7)	7 (8)	78 (17)
Persistent bacteremia	5 (2)	2 (1)	3 (3)	1 (0.2)
Persistent bacteremia ≥3 d	1 (0.4)	0	1 (1)	1 (0.2)
High-risk *Streptococcus *species^d^	48 (19)	29 (12)	19 (8	109 (24)
No. of bottles of 4 positive on admission				
1	2 (1)	1 (1)	1 (1)	65 (14)
2	5 (2)	1 (1)	4 (5)	19 (4)
3	1 (0.4)	1 (1)	0	8 (2)
4	40 (16)	26 (16)	14 (16)	17 (4)
Culture clearance by day 2	37 (15)	22 (13)	15 (17)	109 (24)
Persistent bacteremia	10 (4)	6 (4)	4 (5)	0
Persistent bacteremia ≥3 d	1 (0.4)	1 (1)	0	0

Abbreviation: IE, infective endocarditis.

^a^ Culture clearance by day 2 is defined by a positive culture in at least 1 bottle on day 1 and no positive culture growth on day 2.

^b^ Persistent bacteremia is defined as blood cultures positive for at least 2 days.

^c^ Low-risk streptococci include *S pneumoniae*, *S pyogenes* (group A *Streptococcus*), *S dysgalactiae* (group C *Streptococcus*), *S agalactiae* (group B *Streptococcus*), and *S viridans *group (*S salivarius*, *S anginosus/constellatus*, and *S thermophilus*).

^d^ High-risk streptococci include *S bovis* (*S gallolyticus, S infantarius* [group D *Streptococcus*]), *S viridans* group (*S mitis/cristatus, S sanguinis, S gordonii, S parasanguinis, S sanguinis, S mutans*), and nutritionally variant streptococci (*Abiotrophia defectiva* and *Granulicatella adiacens*).

For all organisms studied, when only 1 of 4 bottles was positive on admission, the negative LR was 0.12 or less (Table 3). Specifically, the negative LRs for only 1 of 4 blood culture bottles positive on admission were 0.12 (95% CI, 0.03-0.49) for MSSA and 0.10 (95% CI, 0.02-0.40) for MRSA. Clearance of bacteremia by day 2 was less helpful for excluding IE caused by MSSA (negative LR, 0.43; 95% CI, 0.28-0.65) than MRSA (negative LR, 0.24; 95% CI, 0.13-0.42).

**Table 3.** Association of Blood Culture Variables With Pretest Probability of Infective Endocarditis

Variables of interest by organism	Sensitivity, % (95% CI)	Specificity, % (95% CI)	Positive LR (95% CI)	Negative LR (95% CI)
Methicillin-susceptible *Staphylococcus aureus*				
Positive, >1 of 4 bottles positive; negative, 1 of 4 bottles positive	97.53 (91.36-99.70)	21.11 (13.21-30.99)	1.24 (1.10-1.38)	0.12 (0.03-0.49)
Positive, persistent bacteremia; negative, culture clearance by day 2^a^^,^^b^	75.31 (64.47-84.22)	57.78 (46.91-68.12)	1.78 (1.36-2.34)	0.43 (0.28-0.65)
Positive, >1 of 4 bottles positive OR persistent bacteremia; negative, 1 of 4 bottles positive AND culture clearance by day 2	100.00 (95.55-100.00)	18.89 (11.41-28.51)	1.23 (1.12-1.36)	NA^c^
Methicillin-resistant *Staphylococcus aureus*				
Positive, >1 of 4 bottles positive; negative, 1 of 4 bottles positive	96.77 (88.83-99.61)	32.94 (23.13-43.98)	1.44 (1.23-1.69)	0.10 (0.02-0.40)
Positive, persistent bacteremia; negative, culture clearance by day 2	83.87 (72.33-91.98)	68.24 (57.24-77.92)	2.64 (1.90-3.67)	0.24 (0.13-0.42)
Positive, >1 of 4 bottles positive OR persistent bacteremia; negative, 1 of 4 bottles positive AND culture clearance by day 2	96.77 (88.83-99.61)	28.24 (19-39.04)	1.35 (1.17-1.55)	0.11 (0.03-0.47)
*Enterococcus faecalis*				
Positive, >1 of 4 bottles positive; negative, 1 of 4 bottles positive	97.22 (85.47-99.93)	52.75 (42-63.31)	2.06 (1.64-2.57)	0.05 (0.01-0.37)
Positive, persistent bacteremia; negative, culture clearance by day 2	69.44 (51.89-83.65)	89.01 (80.72-94.60)	6.32 (3.39-11.79)	0.34 (0.21-0.56)
Positive, >1 of 4 bottles positive OR persistent bacteremia; negative, 1 of 4 bottles positive AND culture clearance by day 2	97.22 (85.47-99.93)	51.65 (40.93-62.26)	2.01 (1.61-2.50)	0.05 (0.01-0.38)
Low-risk *Streptococcus* species^d^				
Positive, >1 of 4 bottles positive; negative, 1 of 4 bottles positive	96 (79.65-99.90)	33.75 (23.55-45.19)	1.45 (1.22-1.73)	0.12 (0.02-0.83)
Positive, persistent bacteremia; negative, culture clearance by day 2	24 (9.36-45.13)	97.50 (91.26-99.70)	9.60 (2.07-44.60)	0.78 (0.62-0.97)
Positive, >1 of 4 bottles positive OR persistent bacteremia; negative, 1 of 4 bottles positive AND culture clearance by day 2	96 (79.65-99.90)	33.75 (23.55-45.19)	1.45 (1.22-1.73)	0.12 (0.02-0.83)
High-risk *Streptococcus* species^e^				
Positive, >1 of 4 bottles positive; negative, 1 of4 bottles positive	95.83 (85.75-99.49)	59.63 (49.81-68.92)	2.37 (1.88-3.01)	0.07 (0.02-0.27)
Positive, persistent bacteremia; negative, culture clearance by day 2	22.92 (12.03-37.31)	100 (96.67-100)	NA^c^	0.77 (0.66-0.90)
Positive, >1 of 4 bottles positive OR persistent bacteremia; negative, 1 of 4 bottles positive AND culture clearance by day 2	95.83 (85.75-99.49)	59.63 (49.81-68.92)	2.37 (1.88-3.01)	0.07 (0.02-0.27)

Abbreviations: LR, likelihood ratio; NA, not applicable.

^a^ Persistent bacteremia is defined as cultures positive for 2 or more days.

^b^ Culture clearance by day 2 equates to blood cultures being positive for only 1 day.

^c^ Represents a value that could not be calculated due to low or 0 cases that fit the criteria in the control group (eg, no false negatives).

^d^ Low-risk *Streptococcus* species include *S pneumoniae*, *S pyogenes* (group A *Streptococcus*), *S dysgalactiae* (group C *Streptococcus*), *S agalactiae* (group B *Streptococcus*), and *S viridans *group (*S salivarius*, *S anginosus/constellatus*, and *S thermophilus*).

^e^ High-risk *Streptococcus* species include *S bovis* (*S gallolyticus, S infantarius* [group D streptococcus]), *S viridans* group (*S mitis/cristatus, S sanguinis, S gordonii, S parasanguinis, S sanguinis, *and *S mutans*), and nutritionally variant streptococci (*Abiotrophia defectiva* and *Granulicatella adiacens*).

Similarly, for *E faecalis*, the negative LR for 1 of 4 bottles positive on admission was 0.05 (95% CI, 0.01-0.37), and culture clearance by day 2 had a negative LR of 0.34 (95% CI, 0.21-0.56). Persistence of bacteremia for at least 2 days with *E faecalis* resulted in a notable positive LR (6.32; 95% CI, 3.39-11.79).

For low-risk *Streptococcus* species, the negative LR for endocarditis when 1 of 4 bottles was positive on admission was 0.12 (95% CI, 0.02-0.83). Clearance of bacteremia by day 2 had a negative LR of 0.78 (95% CI, 0.62-0.97), whereas persistent bacteremia had a positive LR of 9.60 (95% CI, 2.07-44.60).

For high-risk *Streptococcus* species, the negative LR for 1 of 4 bottles positive on admission was 0.07 (95% CI, 0.02-0.27). Culture clearance by day 2 had a negative LR of 0.77 (95% CI, 0.66-0.90). Two of 4 bottles positive on admission also had a low negative LR of 0.25 (95% CI, 0.10-0.62). The positive LR for persistent bacteremia could not be calculated as 100% of the control cases cleared in 24 hours. In addition, 37 of the high-risk streptococci IE cases (77%) cleared by day 2.

For all organisms, when only 1 of 4 bottles was positive and culture clearance was achieved by day 2, the negative LR ranged from 0.05 (95% CI, 0.01-0.37) to 0.12 (95% CI, 0.02-0.83). The positive LRs for persistence of bacteremia ranged from 1.78 (95% CI, 1.36-2.34) to 9.60 (95% CI, 2.07-44.60) (Table 3). Aside from high-risk streptococci, having 2 bottles positive on admission was not helpful in impacting pretest probability, whereas if both 2 of 4 bottles were positive and there was culture clearance by day 2, the negative LR was 0.13 (95% CI, 0.02-1.02) to 0.25 (95% CI, 0.08-0.80). Moreover, when bacteremia was persistent for 3 or more days, the positive LR ranged from 2.09 (95% CI, 1.43-3.06) to 9.10 (95% CI, 3.65-22.67). Four of 4 bottles positive on admission had useful positive LRs depending on the organism, most notable for *E faecalis* (LR, 4.21; 95% CI, 2.53-7.02) and high-risk *Streptococcus* species (LR, 5.35; 95% CI, 3.39-8.42). Likewise, when 4 of 4 bottles were positive on admission and there was persistent bacteremia, the positive LRs ranged from 1.63 (95% CI, 1.17-2.28) to 8.59 (95% CI, 3.43-21.55), again most notably for *E faecalis* and streptococci (eTable 3 in Supplement 1).

Sensitivity analyses for patients who specifically met Duke criteria for definite IE (eTable 4 in Supplement 1) or for control patients who had negative findings on echocardiogram (eTable 5 in Supplement 1) both showed test characteristics similar to those of the primary analysis. In addition, we studied certain combination cohorts, including 1 of 4 positive blood cultures and culture clearance by day 2, 2 of 4 positive blood cultures and culture clearance by day 2, and 4 of 4 positive blood cultures and persistent bacteremia. The first 2 cohorts had a negative LR of 0.00 (95% CI, 0.00-0.31) to 0.22 (95% CI, 0.03-0.58) for all organisms. The later cohort had positive LRs of 1.53 (95% CI, 1.04-2.27) to 6.75 (95% CI, 2.72-16.75), which were most helpful for MRSA, *E faecalis*, and *Streptococcus* species. Among all IE cases, there was only 1 case that had the combination of 1 of 4 positive blood cultures on admission and persistent bacteremia for 3 days or longer; thus, LRs could not be calculated by organism for this combination. Of note, for all streptococcal species, 97% IE cases and 99% control cases cleared within 2 days, and 100% within 3 days.

## Discussion

The all-comer risk of IE is approximately 1 in 8 for patients with *S aureus* bacteremia.^30,31^ In this case-control study, we found that simple assessments of blood culture results, including the number of bottles positive on admission (assuming at least 2 sets were drawn) and the number of days the cultures remained positive, could accurately and potently adjust the pretest probability of IE.

For example, having only 1 blood culture bottle positive was generally associated with negative LR of approximately 0.1. Given a pretest odds ratio of 1 in 8 for *S aureus*, the pretest probability of having only 1 blood culture bottle positive would be 0.1 in 8, or 1 in 80, a very useful shift in pretest probability, informing diagnostic and empirical therapeutic decision-making. Moreover, for MRSA, culture clearance by day 2 had a negative LR of 0.24, a pretest probability of 0.24 in 8, or 1 in 40, a significant change from the pretest odds. For *E faecalis*, the pretest probability of IE ranges from 1 in 6 to 1 in 8.^9,32,33^ One of 4 bottles positive on admission had a negative LR of 0.05, changing the pretest probability of IE to 1 in 120 or less. If there was persistent bacteremia, then the positive LR was 6.32, which would adjust a 1 in 6 pretest probability to a 1 in 2 pretest probability, a very meaningful shift.

Other tools for altering bayesian probabilities of IE are available. For example, there have been multiple studies published establishing predictive scores for IE, including PREDICT,^12,13^ VIRSTA,^14^ POSITIVE,^15^ and most recently in 2024, SABIER.^16^ The first 3 scoring systems use patient risk factors, predisposing conditions, and phenomena based on Duke criteria (ie, vascular or immunological), but only for *S aureus* bacteremia. The first 3 studies showed how persistence of bacteremia and community-onset bacteremia were more important risk factors than hospital-acquired or nosocomial bacteremia for IE. They did not differentiate between MSSA and MRSA, and they did not include other common pathogens that cause IE.

The PREDICT and VIRSTA scoring systems were specifically used to help clinicians decide on the use of transesophageal echocardiograms.^13^ The POSITIVE scoring system emphasized time to blood culture positivity among 10 hospitals in Sweden that all used the same laboratory, which is not available in our hospital system owing to the resource-heavy nature of this task.^15^ SABIER differentiated itself by using demographic-based variables, risk factors, and initial laboratory values in attempt to be independent of subjective clinical judgment, but it did not incorporate blood culture data. This is challenging to apply at bedside because of its complexity.^16^

There is limited literature published regarding prevalence of IE in *E faecalis* bacteremia. One example is the study by Dahl et al,^9^ which reported that 3 or more blood culture–positive bottles was associated with an increased odds of IE; however, it did not analyze persistence of bacteremia. NOVA and DENOVA are 2 IE-predictive scoring systems used for *E faecalis*.^17,18^ The updated versions of these scoring systems utilize a number of positive cultures greater than or equal to 2, but do not include persistent bacteremia. Finally, a few studies have analyzed *Streptococcus* species to create algorithms or scoring systems (eg, HANDOC) on when to obtain a transthoracic echocardiogram and/or transesophageal echocardiogram.^19,26,27,28^ These scoring systems include number of initial positive blood cultures, but did not comment on persistence of bacteremia.

Thus, current prediction scores are complex, may not be facile for clinicians to implement at the bedside, apply to specific individual pathogens, and have a wide range of sensitivity, specificity, and negative predictive value.^34^ There remains uncertainty in the group of patients that only have 1 positive blood culture bottle on admission for a high-risk organism or have rapid culture clearance. None of the aforementioned studies compared the number of positive blood culture bottles on initial presentation as an independent prognostic tool for IE. Our study shows how aspects of several of these scoring systems can be useful on their own, obviating the need for complex pathogen-specific scoring systems.

We found not only that the number of positive blood culture bottles on presentation and days of bacteremia are associated with one’s pretest probability for IE, but also that these associations varied by pathogen (ie, MSSA, MRSA, *E faecalis*, and low-risk and high-risk streptococcal species). Both factors can be used within the first 48 to 72 hours of a patient’s hospitalization to help guide further diagnostics, antibiotic duration, and reduce hospital length of stay. All species had significant negative LRs if only 1 of 4 blood culture bottles was positive on admission. This includes *S aureus*, challenging the current practice that every patient with *S aureus* community-acquired bacteremia requires a transthoracic echocardiogram for further work-up.^1,3,35^ All organisms except streptococci had statistically significant negative LRs if blood cultures cleared by day 2. It was also evident that, as we would expect, if bacteremia persisted for 2 or more days, the positive LRs for IE were significant. Of note, for all streptococcal species, owing to the nature of the organism itself, 97% IE cases and 99% control cases cleared within 2 days, and 100% within 3 days.

### Limitations

The primary limitation of our study is its retrospective nature, which makes it prone to various forms of bias, such as selection, information, and confounding bias. However, prospective evaluation of such criteria is complex to design and implement. Also, not all control patients underwent echocardiography for IE evaluation, which we believe reflects a pragmatic clinical practice utilization of resources based on clinical suspicion. Moreover, our subanalysis for these patients had results similar to those for all cases. It was also not surprising that the IE cohort had more individuals with prosthetic valves and history of injection drug use, because these are known risk factors for IE and are incorporated in the Duke criteria. Furthermore, the use of data from multiple sites, the relatively large sample size, and the detailed nature of the medical record reviews, including multiple individuals verifying the information, provide reassurance that the findings are valid and generalizable.

## Conclusions

In this case-control study of patients with and without IE, we report that the number of bottles positive on admission to the hospital, and the number of days to clearance of bacteremia, are associated with the pretest probability of IE. These simple bedside tests can therefore be used to inform diagnostic and empirical therapeutic management. Specifically, having only 1 of 4 positive blood cultures on admission had a negative LR of approximately 0.1 across pathogens for IE. For streptococci, blood cultures virtually always cleared by day 2, so clearing quickly is not helpful. In contrast, for MSSA, MRSA, and *E faecalis*, clearance of blood cultures after 1 day of therapy was also very helpful at negatively adjusting the risk of IE, whereas persistence of bacteremia resulted in helpful positive LRs.

## Notes

Supplement 1. **eTable 1.** Control Case Breakdown by Echocardiogram **eTable 2.** Diagnoses of Control Cases with Bacteremia for >72 hours **eTable 3.** Influence of Blood Culture Variables on Pre-test Probability of: Infective Endocarditis, Secondary Outcomes (all cases included) **eTable 4.** Influence of Blood Culture Variables on Pre-test Probability of Definite Infective Endocarditis Cases **eTable 5.** Influence of Blood Culture Variables on Pre-test Probability of Definite Infective Endocarditis Cases and Control Cases with Negative Echocardiograms

Supplement 2.
Data Sharing Statement
