# Supplementary material for: Interpreting Blood Culture Results as Early Guidance for Infective Endocarditis
Source: JAMA Netw Open. 2025 May 1;8(5):e258079. doi: 10.1001/jamanetworkopen.2025.8079 (PMC12046426; doi:10.1001/jamanetworkopen.2025.8079)
Supplement: Supplement 1. — eTable 1. Control Case Breakdown by Echocardiogram eTable 2. Diagnoses of Control Cases with Bacteremia for >72 hours eTable 3. Influence of Blood Culture Variables on Pre-test Probability of: Infective Endocarditis, Secondary Outcomes (all cases included) eTable 4. Influence of Blood Culture Variables on Pre-test Probability of Definite Infective Endocarditis Cases eTable 5. Influence of Blood Culture Variables on Pre-test Probability of Definite Infective Endocarditis Cases and Control Cases with Negative Echocardiograms [file jamanetwopen-e258079-s001.pdf]

Supplemental Online Content

Freling SR, Richie I, Norwitz D, et al. Interpreting blood culture results as early guidance for infective endocarditis. *JAMA Netw Open*. 2025;8(5):e258079. doi:10.1001/jamanetworkopen.2025.8079

eTable 1. Control Case Breakdown by Echocardiogram
eTable 2. Diagnoses of Control Cases with Bacteremia for >72 hours
eTable 3. Influence of Blood Culture Variables on Pre-test Probability of: Infective Endocarditis, Secondary Outcomes (all cases included)
eTable 4. Influence of Blood Culture Variables on Pre-test Probability of Definite Infective Endocarditis Cases
eTable 5. Influence of Blood Culture Variables on Pre-test Probability of Definite Infective Endocarditis Cases and Control Cases with Negative Echocardiograms

This supplemental material has been provided by the authors to give readers additional information about their work.

© 2025 Freling SR et al. *JAMA Network Open*.

eTable 1. Control Case Breakdown by Echocardiogram

	Number of Cases (%)	Negative TTE or TEE + Low clinical suspicion for IE (%)	No clinical suspicion for IE (%)
**MSSA (90)**	90 (20)	82 (18)	8 (2)
**MRSA (85)**	85 (19)	67 (15)	18 (4)
***E. faecalis* (91)**	91 (20)	63 (14)	28 (6)
**Total *Strep* spp. (189)**	189 (41)	132 (29)	57 (13)
**Low-risk Streptococci[a] (80)**	80 (18)	53 (12)	27 (6)
**High-risk Streptococci[b] (109)**	109 (24)	79 (17)	30 (7)
**Total Control Cases**	455	344 (76)	111 (24)

Data are presented as number (No/total No of control cases %).1/4 to 4/4 = number of bottles positive on admission out of four.
a = Low Risk Streptococci: *S. pneumoniae*, *S. pyogenes* (GAS), *S. dysgalactiae* (GCS), *S. agalactiae* (GBS), *S. viridans group* (*S. salivarius*, *S. anginosus/constellatus*, *S. thermophilus*)
b = High Risk Streptococci: *S. bovis* (*S. gallolyticus, S. infantarius* (GDS)), *S. viridans* group (*S. mitis/cristatus, S. sanguinis, S. gordonii, S. parasanguinis, S. sanguinis, S. mutans*), Nutritionally variant strep (NVS): *Abiotrophia defectiva*, *Granulicatella adiacens*
Abbreviations: IE = infective endocarditis, MSSA = methicillin-susceptible *Staphylococcus aureus*, MRSA = methicillin-resistant *Staphylococcus aureus*.

© 2025 Freling SR et al. *JAMA Network Open*.

eTable 2. Diagnoses of Control Cases with Bacteremia for >72 hours

	Total Cases	Bone/Skeletal Disease[α]	Cather-related infection	Deep/Necrotizing Soft Tissue Infection	Subdural empyema	Intra-abdominal infection
**MSSA**	13	7	5	1	-	-
**MRSA**	14	7	3	3	1	-
E. faecalis	3	1	-	-	-	2
***Streptococcus* spp.**	-	-	-	-	-	-

α = e.g., septic arthritis, osteomyelitis, abscess
- = no episodes in that group
Abbreviations: MSSA = methicillin-susceptible *Staphylococcus aureus*, MRSA = methicillin-resistant *Staphylococcus aureus, E. faecalis* = *Enterococcus faecalis*

© 2025 Freling SR et al. *JAMA Network Open*.

eTable 3. Influence of Blood Culture Variables on Pre-test Probability of: Infective Endocarditis, Secondary Outcomes (all cases included)

Variables of Interest by Organism		Sensitivity (%) (95% CI)	Specificity (%) (95% CI)	+LR (95% CI)	-LR (95% CI)
**MSSA**					
**Positive: ≥3/4 bottles positive**	**Negative: 2/4 bottles positive**	92.41 (84.20 to 97.16)	11.27 (4.99 to 21.00)	1.04 (0.94 to 1.16)	0.67 (0.25 to 1.85)
**Positive: 4/4 bottles positive**	**Negative: ≤3/4 bottles positive**	79.01 (68.54 to 87.27)	41.11 (30.84 to 51.98)	1.34 (1.09 to 1.65)	0.51 (0.31 to 0.83)
**Positive: Persistent bacteremia ≥ 3 days**	**Negative: Culture clearance by day 3**	58.02 (46.54 to 68.91)	72.22 (61.78 to 81.15)	2.09 (1.43 to 3.06)	0.58 (0.44 to 0.77)
**Positive: >2/4 bottles positive OR persistent bacteremia[a]**	**Negative: 2/4 bottles positive AND Culture clearance by day 2[b]**	98.77 (93.31 to 99.97)	9.59 (3.94 to 18.76)	1.09 (1.01 to 1.18)	0.13 (0.02 to 1.02)
**Positive: 4/4 bottles positive AND persistent bacteremia**	**Negative: ≤3/4 bottles positive OR culture clearance by day 2**	58.02 (46.54 to 68.91)	64.44 (53.65 to 74.26)	1.63 (1.17 to 2.28)	0.65 (0.48 to 0.88)
**MRSA**					
**Positive: ≥3/4 bottles positive**	**Negative: 2/4 bottles positive**	73.33 (60.34 to 83.93)	33.33 (21.40 to 47.06)	1.10 (0.87 to 1.40)	0.80 (0.46 to 1.40)
**Positive: 4/4 bottles positive**	**Negative: ≤3/4 bottles positive**	64.52 (51.34 to 76.26)	65.88 (54.80 to 75.82)	1.89 (1.33 to 2.68)	0.54 (0.37 to 0.78)
**Positive: Persistent bacteremia ≥ 3 days**	**Negative: Culture clearance by day 3**	80.65 (68.63 to 89.58)	74.12 (63.48 to 83.01)	3.12 (2.13 to 4.56)	0.26 (0.15 to 0.44)
**Positive: >2/4 bottles positive OR persistent bacteremia**	**Negative: 2/4 bottles positive AND Culture clearance by day 2**	95.00 (86.08 to 98.96)	26.23 (15.80 to 39.07)	1.29 (1.10 to 1.51)	0.19 (0.06 to 0.62)
**Positive: 4/4 bottles positive AND persistent bacteremia**	**Negative: ≤3/4 bottles positive OR culture clearance by day 2**	58.06 (44.85 to 70.49)	78.82 (68.61 to 86.94)	2.74 (1.73 to 4.35)	0.53 (0.39 to 0.73)
E. faecalis					
**Positive: ≥3/4 bottles positive**	**Negative: 2/4 bottles positive**	77.14 (59.86 to 89.58)	37.21 (22.98 to 53.27)	1.23 (0.92 to 1.65)	0.61 (0.30 to 1.26)
**Positive: 4/4 bottles positive**	**Negative: ≤3/4 bottles positive**	69.44 (51.89 to 83.65)	83.52 (74.27 to 90.47)	4.21 (2.53 to 7.02)	0.37 (0.22 to 0.60)
**Positive: Persistent bacteremia ≥ 3 days**	**Negative: Culture clearance by day 3**	50.00 (32.92 to 67.08)	94.51 (87.64 to 98.19)	9.10 (3.65 to 22.67)	0.53 (0.38 to 0.74)
**Positive: >2/4 bottles positive OR**	**Negative: 2/4 bottles positive AND**	91.43 (76.94 to 98.20)	34.09 (20.49 to 49.92)	1.39 (1.10 to 1.76)	0.25 (0.08 to 0.80)

© 2025 Freling SR et al. *JAMA Network Open*.

**persistent bacteremia**	**Culture clearance by day 2**				
**Positive: 4/4 bottles positive AND persistent bacteremia**	**Negative: ≤3/4 bottles positive OR culture clearance by day 2**	47.22 (30.41 to 64.51)	94.51 (87.64 to 98.19)	8.59 (3.43 to 21.55)	0.56 (0.41 to 0.76)
**Low-risk *Streptococcus* spp.[b]**					
**Positive: ≥3/4 bottles positive**	**Negative: 2/4 bottles positive**	91.67 (73.00 to 98.97)	18.87 (9.44 to 31.97)	1.13 (0.95 to 1.35)	0.44 (0.10 to 1.86)
**Positive: 4/4 bottles positive**	**Negative: ≤3/4 bottles positive**	84.00 (63.92 to 95.46)	58.75 (47.18 to 69.65)	2.04 (1.49 to 2.78)	0.27 (0.11 to 0.68)
**Positive: Persistent bacteremia ≥ 3 days**	**Negative: Culture clearance by day 3**	4.00 (0.10 to 20.35)	98.75 (93.23 to 99.97)	3.20 (0.21 to 49.32)	0.97 (0.89 to 1.06)
**Positive: >2/4 bottles positive OR persistent bacteremia**	**Negative: 2/4 bottles positive AND Culture clearance by day 2**	95.83 (78.88 to 99.89)	18.87 (9.44 to 31.97)	1.18 (1.01 to 1.38)	0.22 (0.03 to 1.63)
**Positive: 4/4 bottles positive AND persistent bacteremia**	**Negative: ≤3/4 bottles positive OR culture clearance by day 2**	17.24 (5.85 to 35.77)	97.50 (91.26 to 99.70)	6.90 (1.42 to 33.61)	0.85 (0.72 to 1.01)
**High-risk *Streptococcus* spp.[c]**					
**Positive: ≥3/4 bottles positive**	**Negative: 2/4 bottles positive**	89.13 (76.43 to 96.38)	43.18 (28.35 to 58.97)	1.57 (1.19 to 2.07)	0.25 (0.10 to 0.62)
**Positive: 4/4 bottles positive**	**Negative: ≤3/4 bottles positive**	83.33 (69.78 to 92.52)	84.40 (76.21 to 90.64)	5.35 (3.39 to 8.42)	0.20 (0.10 to 0.37)
**Positive: Persistent bacteremia ≥ 3 days**	**Negative: Culture clearance by day 3**	2.08 (0.05 to 11.07)	100 (96.67 to 100)	NA	0.97 (0.92 to 1.03)
**Positive: >2/4 bottles positive OR persistent bacteremia**	**Negative: 2/4 bottles positive AND Culture clearance by day 2**	89.13 (76.43 to 96.38)	43.18 (28.35 to 58.97)	1.57 (1.19 to 2.07)	0.25 (0.10 to 0.62)
**Positive: 4/4 bottles positive AND persistent bacteremia**	**Negative: ≤3/4 bottles positive OR culture clearance by day 2**	22.92 (12.03 to 37.31)	100 (96.67 to 100)	NA	0.77 (0.66 to 0.90)

An unfilled box (NA) represents a value which could not be calculated due to low or 0 cases that fit the criteria in the control group (e.g., no false negatives).
a = persistent bacteremia defined as positive cultures for two days, unless otherwise specified above.
b = Low Risk Streptococci: *S. pneumoniae*, *S. pyogenes* (GAS), *S. dysgalactiae* (GCS), *S. agalactiae* (GBS), *S. viridans group* (*S. salivarius*, *S. anginosus/constellatus*, *S. thermophilus*)
c = High Risk Streptococci: *S. bovis* (*S. gallolyticus, S. infantarius* (GDS)), *S. viridans* group (*S. mitis/cristatus, S. sanguinis, S. gordonii, S. parasanguinis, S. sanguinis, S. mutans*), Nutritionally variant strep (NVS): *Abiotrophia defectiva*, *Granulicatella adiacens*
Abbreviations: +LR = positive likelihood ratio; -LR = negative likelihood ratio; CI = confidence interval, NA = not available, MSSA = methicillin-susceptible *Staphylococcus aureus*, MRSA = methicillin-resistant *Staphylococcus aureus, E. faecalis = Enterococcus faecalis*

© 2025 Freling SR et al. *JAMA Network Open*.

eTable 4. Influence of Blood Culture Variables on Pre-test Probability of Definite Infective Endocarditis Cases

Variables of Interest by Organism		Sensitivity (%)<br>(95% CI)	Specificity (%)<br>(95% CI)	+LR<br>(95% CI)	-LR<br>(95% CI)
**MSSA**					
**Positive:**<br>**>1/4 bottles positive**	**Negative:**<br>**1/4 bottles positive**	97.90<br>(88.93 to 99.95)	21.11<br>(13.21 to 30.99)	1.24<br>(1.11 to 1.39)	0.10<br>(0.01 to 0.71)
**Positive:**<br>**≥3/4 bottles positive**	**Negative:**<br>**2/4 bottles positive**	93.62<br>(82.46 to 98.66)	11.27<br>(4.99 to 21.00)	1.06<br>(0.94 to 1.18)	0.57<br>(0.16 to 2.03)
**Positive:**<br>**4/4 bottles positive**	**Negative:**<br>**≤3/4 bottles positive**	81.25<br>(67.37 to 91.05)	41.11<br>(30.84 to 51.98)	1.38<br>(1.11 to 1.72)	0.46<br>(0.24 to 0.86)
**Positive:**<br>**Persistent bacteremia**[a]	**Negative:**<br>**Culture clearance by day 2**[b]	68.75<br>(53.75 to 81.34)	57.78<br>(46.91 to 68.12)	1.63<br>(1.20 to 2.22)	0.54<br>(0.34 to 0.85)
**Positive:**<br>**Persistent bacteremia ≥ 3 days**	**Negative:**<br>**Culture clearance by day 3**	47.27<br>(33.65 to 61.20)	72.22<br>(61.78 to 81.15)	1.70<br>(1.10 to 2.63)	0.73<br>(0.55 to 0.97)
**Positive**:<br>**>1/4 bottles positive OR persistent bacteremia**	**Negative:**<br>**1/4 bottles positive AND culture clearance by day 2**	100<br>(92.60 to 100)	18.89<br>(11.41 to 28.51)	1.23<br>(1.12 to 1.36)	0.00<br>(0.00 to 0.23)
**Positive:**<br>**>2/4 bottles positive OR persistent bacteremia**	**Negative:**<br>**2/4 bottles positive AND Culture clearance by day 2**	97.92<br>(88.93 to 99.95)	9.59<br>(3.94 to 18.76)	1.08<br>(0.99 to 1.18)	0.22<br>(0.03 to 1.71)
**Positive:**<br>**4/4 bottles positive AND persistent bacteremia**	**Negative:**<br>**≤3/4 bottles positive OR culture clearance by day 2**	54.17<br>(39.17 to 68.63)	64.44<br>(53.65 to 74.26)	1.52<br>(1.04 to 2.23)	0.71<br>(0.50 to 1.00)
**MRSA**					
**Positive:**<br>**>1/4 bottles positive**	**Negative:**<br>**1/4 bottles positive**	100<br>(92.29 to 100)	32.94<br>(23.13 to 43.98)	1.49<br>(1.28 to 1.73)	0.00<br>(0.00 to 0.49)
**Positive:**<br>**≥3/4 bottles positive**	**Negative:**<br>**2/4 bottles positive**	71.74<br>(56.54 to 84.01)	33.33<br>(21.40 to 47.06)	1.08<br>(0.83 to 1.39)	0.85<br>(0.47 to 1.53)
**Positive:**<br>**4/4 bottles positive**	**Negative:**<br>**≤3/4 bottles positive**	65.22<br>(49.75 to 78.65)	65.88<br>(54.80 to 75.82)	1.91<br>(1.33 to 2.75)	0.53<br>(0.35 to 0.81)
**Positive:**<br>**Persistent bacteremia**	**Negative:**<br>**Culture clearance by day 2**	86.96<br>(73.74 to 95.06)	68.24<br>(57.24 to 77.92)	2.74<br>(1.97 to 3.81)	0.19<br>(0.09 to 0.41)
**Positive:**<br>**Persistent bacteremia ≥ 3 days**	**Negative:**<br>**Culture clearance by day 3**	82.98<br>(69.19 to 92.35)	74.12<br>(63.48 to 83.01)	3.21<br>(2.19 to 4.70)	0.23<br>(0.12 to 0.44)
**Positive**:<br>**>1/4 bottles positive OR persistent bacteremia**	**Negative:**<br>**1/4 bottles positive AND culture clearance by day 2**	100<br>(92.29 to 100)	28.24<br>(19.00 to 39.04)	1.39<br>(1.22 to 1.59)	0.00<br>(0.00 to 0.39)
**Positive:**<br>**>2/4 bottles positive OR persistent bacteremia**	**Negative:**<br>**2/4 bottles positive AND Culture clearance by day 2**	93.48<br>(82.10 to 98.63)	26.23<br>(15.80 to 39.07)	1.27<br>(1.07 to 1.50)	0.25<br>(0.08 to 0.80)

© 2025 Freling SR et al. *JAMA Network Open*.

**Positive:<br>4/4 bottles positive AND persistent bacteremia**	**Negative:<br>≤3/4 bottles positive OR culture clearance by day 2**	60.87<br>(45.37 to 74.91)	78.82<br>(68.61 to 86.94)	2.87<br>(1.79 to 4.60)	0.50<br>(0.34 to 0.72)
E. faecalis					
**Positive:<br>>1/4 bottles positive**	**Negative:<br>1/4 bottles positive**	96.43<br>(81.65 to 99.91)	52.75<br>(42.00 to 63.31)	2.04<br>(1.62 to 2.56)	0.07<br>(0.01 to 0.47)
**Positive:<br>≥3/4 bottles positive**	**Negative:<br>2/4 bottles positive**	77.78<br>(57.74 to 91.38)	37.21<br>(22.98 to 53.27)	1.24<br>(0.91 to 1.68)	0.60<br>(0.27 to 1.34)
**Positive:<br>4/4 bottles positive**	**Negative:<br>≤3/4 bottles positive**	75.00<br>(55.13 to 89.31)	83.52<br>(74.27 to 90.57)	4.55<br>(2.73 to 7.57)	0.30<br>(0.16 to 0.57)
**Positive:<br>Persistent bacteremia**	**Negative:<br>Culture clearance by day 2**	78.57<br>(59.05 to 91.70)	89.01<br>(80.72 to 94.60)	7.15<br>(3.86 to 13.24)	0.24<br>(0.12 to 0.49)
**Positive:<br>Persistent bacteremia ≥ 3 days**	**Negative:<br>Culture clearance by day 3**	56.67<br>(37.43 to 74.54)	94.51<br>(87.64 to 98.19)	10.31<br>(4.16 to 25.56)	0.46<br>(0.30 to 0.69)
**Positive**:<br>**>1/4 bottles positive OR persistent bacteremia**	**Negative:<br>1/4 bottles positive AND culture clearance by day 2**	96.43<br>(81.65 to 99.91)	51.65<br>(40.93 to 62.26)	2.01<br>(1.61 to 2.50)	0.05<br>(0.01 to 0.38)
**Positive:<br>>2/4 bottles positive OR persistent bacteremia**	**Negative:<br>2/4 bottles positive AND Culture clearance by day 2**	96.30<br>(81.03 to 99.91)	34.09<br>(20.49 to 49.92)	1.46<br>(1.17 to 1.83)	0.11<br>(0.02 to 0.78)
**Positive:<br>4/4 bottles positive AND persistent bacteremia**	**Negative:<br>≤3/4 bottles positive OR culture clearance by day 2**	60.71<br>(40.58 to 78.50)	94.51<br>(87.64 to 98.19)	11.05<br>(4.48 to 27.25)	0.42<br>(0.41 to 0.76)
**Low-risk *Streptococcus* spp.[c]**					
**Positive:<br>>1/4 bottles positive**	**Negative:<br>1/4 bottles positive**	100<br>(76.84 to 100)	33.75<br>(23.55 to 45.19)	1.51<br>(1.29 to 1.76)	0.00<br>(0.00 to 0.51)
**Positive:<br>≥3/4 bottles positive**	**Negative:<br>2/4 bottles positive**	100<br>(76.84 to 100)	18.87<br>(9.44 to 31.97)	1.23<br>(1.08 to 1.40)	0.00<br>(0.00 to 0.23)
**Positive:<br>4/4 bottles positive**	**Negative:<br>≤3/4 bottles positive**	100<br>(76.84 to 100)	58.75<br>(47.18 to 69.65)	2.42<br>(1.87 to 3.15)	0.00<br>(0.00 to 1.42)
**Positive:<br>Persistent bacteremia**	**Negative:<br>Culture clearance by day 2**	14.29<br>(1.78 to 42.81)	97.50<br>(91.26 to 99.70)	5.71<br>(0.88 to 37.30)	0.88<br>(0.71 to 1.09)
**Positive:<br>Persistent bacteremia ≥ 3 days**	**Negative:<br>Culture clearance by day 3**	0.00<br>(0.00 to 19.51)	98.75<br>(93.23 to 99.97)	0.00<br>(0.00 to 3.00)	1.01<br>(0.99 to 1.04)
**Positive**:<br>**>1/4 bottles positive OR persistent bacteremia**	**Negative:<br>1/4 bottles positive AND culture clearance by day 2**	100<br>(76.84 to 100)	33.75<br>(23.55 to 45.19)	1.51<br>(1.29 to 1.76)	0.00<br>(0.00 to 0.51)
**Positive:<br>>2/4 bottles positive OR**	**Negative:<br>2/4 bottles positive AND**	100<br>(76.84 to 100)	18.87<br>(9.44 to 31.97)	1.23<br>(1.08 to 1.40)	0.00<br>(0.00 to 0.23)

© 2025 Freling SR et al. *JAMA Network Open*.

persistent bacteremia	Culture clearance by day 2				
**Positive:** 4/4 bottles positive AND persistent bacteremia	**Negative:** ≤3/4 bottles positive OR culture clearance by day 2	14.29 (1.78 to 42.81)	97.50 (91.26 to 99.70)	5.71 (0.88 to 37.30)	0.88 (0.71 to 1.09)
**High-risk *Streptococcus* spp.[d]**					
**Positive:** >1/4 bottles positive	**Negative:** 1/4 bottles positive	96.55 (82.24 to 99.91)	59.63 (49.81 to 68.92)	2.39 (1.88 to 3.04)	0.06 (0.01 to 0.40)
**Positive:** ≥3/4 bottles positive	**Negative:** 2/4 bottles positive	96.43 (81.65 to 99.91)	43.18 (28.35 to 58.97)	1.7 (1.30 to 2.22)	0.08 (0.01 to 0.58)
**Positive:** 4/4 bottles positive	**Negative:** ≤3/4 bottles positive	89.66 (72.65 to 97.81)	84.40 (76.21 to 90.64)	5.75 (3.65 to 9.05)	0.12 (0.04 to 0.36)
**Positive:** Persistent bacteremia	**Negative:** Culture clearance by day 2	NA	NA	NA	NA
**Positive:** Persistent bacteremia ≥ 3 days	**Negative:** Culture clearance by day 3	NA	NA	NA	NA
**Positive:** >1/4 bottles positive OR persistent bacteremia	**Negative:** 1/4 bottles positive AND culture clearance by day 2	96.55 (82.24 to 99.91)	59.63 (49.81 to 68.92)	2.39 (1.88 to 3.04)	0.06 (0.01 to 0.40)
**Positive:** >2/4 bottles positive OR persistent bacteremia	**Negative:** 2/4 bottles positive AND Culture clearance by day 2	96.43 (81.65 to 99.91)	43.18 (28.35 to 58.97)	1.7 (1.30 to 2.22)	0.08 (0.01 to 0.58)
**Positive:** 4/4 bottles positive AND persistent bacteremia	**Negative:** ≤3/4 bottles positive OR culture clearance by day 2	24.14 (10.30 to 43.54)	100 (96.67 to 100)	NA	0.76 (0.62 to 0.93)

An unfilled box (NA) represents a value which could not be calculated due to low or 0 cases that fit the criteria in the control group (e.g., no false negatives).
a = persistent bacteremia is defined as cultures positive for two or more days, unless otherwise specified above.
b = culture clearance by day 2 equates to blood cultures being positive for only one day
c = Low Risk Streptococci: *S. pneumoniae*, *S. pyogenes* (GAS), *S. dysgalactiae* (GCS), *S. agalactiae* (GBS), *S. viridans group* (*S. salivarius*, *S. anginosus/constellatus*, *S. thermophilus*)
d = High Risk Streptococci: *S. bovis* (*S. gallolyticus, S. infantarius* (GDS)), *S. viridans* group (*S. mitis/cristatus, S. sanguinis, S. gordonii, S. parasanguinis, S. sanguinis, S. mutans*), Nutritionally variant strep (NVS): *Abiotrophia defectiva*, *Granulicatella adiacens*
Abbreviations: +LR = positive likelihood ratio; -LR = negative likelihood ratio; CI = confidence interval, NA = not available, MSSA = methicillin-susceptible *Staphylococcus aureus*, MRSA = methicillin-resistant *Staphylococcus aureus, E. faecalis = Enterococcus faecalis*

© 2025 Freling SR et al. *JAMA Network Open*.

eTable 5. Influence of Blood Culture Variables on Pre-test Probability of Definite Infective Endocarditis Cases and Control Cases with Negative Echocardiograms

Variables of Interest by Organism		Sensitivity (%) (95% CI)	Specificity (%) (95% CI)	+LR (95% CI)	-LR (95% CI)
**MSSA**					
**Positive: >1/4 bottles positive**	**Negative: 1/4 bottles positive**	97.92 (88.93 to 99.95)	20.73 (12.57 to 31.11)	1.24 (1.10 to 1.39)	0.10 (0.01 to 0.73)
**Positive: ≥3/4 bottles positive**	**Negative: 2/4 bottles positive**	93.62 (82.46 to 98.66)	9.23 (3.46 to 19.02)	1.03 (0.93 to 1.15)	0.69 (0.18 to 2.63)
**Positive: 4/4 bottles positive**	**Negative: ≤3/4 bottles positive**	81.25 (67.37 to 91.05)	39.02 (28.44 to 50.43)	1.33 (1.07 to 1.66)	0.48 (0.25 to 0.92)
**Positive: Persistent bacteremia[a]**	**Negative: Culture clearance by day 2[b]**	68.75 (53.75 to 81.34)	57.32 (45.91 to 68.18)	1.61 (1.18 to 2.21)	0.55 (0.34 to 0.86)
**Positive: Persistent bacteremia ≥ 3 days**	**Negative: Culture clearance by day 3**	47.27 (33.65 to 61.20)	72.22 (61.78 to 81.15)	1.70 (1.10 to 2.63)	0.73 (0.55 to 0.97)
**Positive**: **>1/4 bottles positive OR persistent bacteremia**	**Negative: 1/4 bottles positive AND culture clearance by day 2**	100 (92.60 to 100)	18.29 (10.62 to 28.37)	1.22 (1.10 to 1.36)	0.00 (0.00 to 0.22)
**Positive: >2/4 bottles positive OR persistent bacteremia**	**Negative: 2/4 bottles positive AND Culture clearance by day 2**	97.92 (88.93 to 99.95)	16.30 (9.42 to 25.46)	1.17 (1.06 to 1.29)	0.13 (0.02 to 0.94)
**Positive: 4/4 bottles positive AND persistent bacteremia**	**Negative: ≤3/4 bottles positive OR culture clearance by day 2**	54.17 (39.17 to 68.63)	64.63 (53.30 to 74.88)	1.53 (1.04 to 2.27)	0.71 (0.50 to 1.00)
**MRSA**					
**Positive: >1/4 bottles positive**	**Negative: 1/4 bottles positive**	100 (92.29 to 100)	28.36 (18.01 to 40.69)	1.40 (1.20 to 1.62)	0.00 (0.00 to 0.40)
**Positive: ≥3/4 bottles positive**	**Negative: 2/4 bottles positive**	71.74 (56.54 to 84.01)	25.00 (13.64 to 39.60)	0.96 (0.75 to 1.22)	1.13 (0.58 to 2.21)
**Positive: 4/4 bottles positive**	**Negative: ≤3/4 bottles positive**	65.22 (49.75 to 78.65)	58.21 (45.52 to 70.15)	1.56 (1.10 to 2.22)	0.60 (0.38 to 0.93)
**Positive: Persistent bacteremia**	**Negative: Culture clearance by day 2**	86.96 (73.74 to 95.06)	62.69 (50.01 to 74.20)	2.33 (1.68 to 3.24)	0.21 (0.10 to 0.45)
**Positive: Persistent bacteremia ≥ 3 days**	**Negative: Culture clearance by day 3**	82.98 (69.19 to 92.35)	74.12 (63.48 to 83.01)	3.21 (2.19 to 4.70)	0.23 (0.12 to 0.44)
**Positive**: **>1/4 bottles positive OR**	**Negative:**	100 (92.29 to 100)	23.88 (14.31 to 35.86)	1.31 (1.15 to 1.50)	0.00 (0.00 to 0.31)

© 2025 Freling SR et al. *JAMA Network Open*.

**persistent bacteremia**	**1/4 bottles positive AND culture clearance by day 2**				
**Positive: >2/4 bottles positive OR persistent bacteremia**	**Negative: 2/4 bottles positive AND Culture clearance by day 2**	93.48 (82.10 to 98.63)	38.81 (27.14 to 51.50)	1.53 (1.24 to 1.88)	0.17 (0.05 to 0.52)
**Positive: 4/4 bottles positive AND persistent bacteremia**	**Negative: ≤3/4 bottles positive OR culture clearance by day 2**	60.87 (45.37 to 74.91)	73.13 (60.90 to 83.24)	2.27 (1.43 to 3.58)	0.54 (0.36 to 0.79)
E. faecalis					
**Positive: >1/4 bottles positive**	**Negative: 1/4 bottles positive**	96.43 (81.65 to 99.91)	49.21 (36.38 to 62.11)	1.90 (1.47 to 2.45)	0.07 (0.01 to 0.51)
**Positive: ≥3/4 bottles positive**	**Negative: 2/4 bottles positive**	77.78 (57.74 to 91.38)	37.50 (21.10 to 56.31)	1.24 (0.89 to 1.74)	0.59 (0.26 to 1.37)
**Positive: 4/4 bottles positive**	**Negative: ≤3/4 bottles positive**	75.00 (55.13 to 59.31)	80.95 (69.09 to 89.31)	3.94 (2.27 to 6.84)	0.31 (0.16 to 0.59)
**Positive: Persistent bacteremia**	**Negative: Culture clearance by day 2**	78.57 (59.05 to 91.70)	84.13 (72.74 to 92.12)	4.95 (2.72 to 9.02)	0.25 (0.12 to 0.52)
**Positive: Persistent bacteremia ≥ 3 days**	**Negative: Culture clearance by day 3**	56.67 (37.43 to 74.54)	94.51 (87.64 to 98.19)	10.31 (4.16 to 25.56)	0.46 (0.30 to 0.69)
**Positive: >1/4 bottles positive OR persistent bacteremia**	**Negative: 1/4 bottles positive AND culture clearance by day 2**	96.43 (81.65 to 99.91)	47.62 (34.88 to 60.59)	1.84 (1.44 to 2.35)	0.07 (0.01 to 0.52)
**Positive: >2/4 bottles positive OR persistent bacteremia**	**Negative: 2/4 bottles positive AND Culture clearance by day 2**	96.30 (81.03 to 99.91)	17.46 (9.05 to 29.10)	1.17 (1.02 to 1.34)	0.21 (0.03 to 1.56)
**Positive: 4/4 bottles positive AND persistent bacteremia**	**Negative: ≤3/4 bottles positive OR culture clearance by day 2**	53.57 (33.87 to 72.49)	92.06 (82.44 to 97.37)	6.75 (2.72 to 16.75)	0.50 (0.34 to 0.76)
**Low-risk *Streptococcus* spp.[c]**					
**Positive: >1/4 bottles positive**	**Negative: 1/4 bottles positive**	100 (76.84 to 100)	28.30 (16.79 to 42.35)	1.39 (1.18 to 1.65)	0.00 (0.00 to 0.40)
**Positive: ≥3/4 bottles positive**	**Negative: 2/4 bottles positive**	100 (76.84 to 100)	15.79 (6.02 to 31.25)	1.19 (1.03 to 1.36)	0.00 (0.00 to 0.19)
**Positive: 4/4 bottles positive**	**Negative: ≤3/4 bottles positive**	100 (78.84 to 100)	54.72 (40.45 to 68.44)	2.21 (1.64 to 2.97)	0.00 (0.00 to 1.21)
**Positive: Persistent bacteremia**	**Negative: Culture clearance by day 2**	14.29 (1.78 to 42.81)	96.23 (87.02 to 99.54)	3.79 (0.58 to 24.55)	0.89 (0.71 to 1.11)
**Positive:**	**Negative:**	0.00	98.75	0.00	1.01

© 2025 Freling SR et al. *JAMA Network Open*.

**Persistent bacteremia ≥ 3 days**	**Culture clearance by day 3**	(0.00 to 19.51)	(93.23 to 99.97)	(0.00 to 2.88)	(0.99 to 1.04)
**Positive**:<br>**>1/4 bottles positive OR persistent bacteremia**	**Negative:**<br>**1/4 bottles positive AND culture clearance by day 2**	100<br>(76.84 to 100)	28.30<br>(16.79 to 42.35)	1.39<br>(1.18 to 1.65)	0.00<br>(0.00 to 0.40)
**Positive:**<br>**>2/4 bottles positive OR persistent bacteremia**	**Negative:**<br>**2/4 bottles positive AND Culture clearance by day 2**	100<br>(76.84 to 100)	11.32<br>(4.27 to 23.03)	1.13<br>(1.02 to 1.24)	0.00<br>(0.00 to 0.13)
**Positive:**<br>**4/4 bottles positive AND persistent bacteremia**	**Negative:**<br>**≤3/4 bottles positive OR culture clearance by day 2**	14.39<br>(1.78 to 42.81)	96.23<br>(87.02 to 99.54)	3.79<br>(0.58 to 24.55)	0.89<br>(0.71 to 1.11)
**High-risk *Streptococcus* spp.[d]**					
**Positive:**<br>**>1/4 bottles positive**	**Negative:**<br>**1/4 bottles positive**	96.55<br>(82.24 to 99.91)	55.70<br>(44.08 to 66.88)	2.18<br>(1.69 to 2.82)	0.06<br>(0.01 to 0.43)
**Positive:**<br>**≥3/4 bottles positive**	**Negative:**<br>**2/4 bottles positive**	96.43<br>(81.65 to 99.91)	37.14<br>(21.47 to 55.08)	1.53<br>(1.18 to 2.00)	0.10<br>(0.01 to 0.69)
**Positive:**<br>**4/4 bottles positive**	**Negative:**<br>**≤3/4 bottles positive**	89.66<br>(72.65 to 97.81)	79.75<br>(69.20 to 87.96)	4.43<br>(2.81 to 6.98)	0.13<br>(0.04 to 0.38)
**Positive:**<br>**Persistent bacteremia**	**Negative:**<br>**Culture clearance by day 2**	24.14<br>(10.30 to 43.54)	100<br>(95.44 to 100)	NA	0.76<br>(0.62 to 0.93)
**Positive:**<br>**Persistent bacteremia ≥ 3 days**	**Negative:**<br>**Culture clearance by day 3**	NA	NA	NA	NA
**Positive**:<br>**>1/4 bottles positive OR persistent bacteremia**	**Negative:**<br>**1/4 bottles positive AND culture clearance by day 2**	96.55<br>(82.24 to 99.91)	55.70<br>(44.08 to 66.88)	2.18<br>(1.69 to 2.82)	0.06<br>(0.01 to 0.43)
**Positive:**<br>**>2/4 bottles positive OR persistent bacteremia**	**Negative:**<br>**2/4 bottles positive AND Culture clearance by day 2**	96.43<br>(81.65 to 99.91)	16.46<br>(9.06 to 26.49)	1.15<br>(1.02 to 1.30)	0.22<br>(0.03 to 1.58)
**Positive:**<br>**4/4 bottles positive AND persistent bacteremia**	**Negative:**<br>**≤3/4 bottles positive OR culture clearance by day 2**	21.14<br>(10.30 to 43.54)	100<br>(95.44 to 100)	NA	0.76<br>(0.62 to 0.93)

An unfilled box (NA) represents a value which could not be calculated due to low or 0 cases that fit the criteria in the control group (e.g., no false negatives).
a = persistent bacteremia is defined as cultures positive for two or more days, unless otherwise specified above.
b = culture clearance by day 2 equates to blood cultures being positive for only one day
c = Low Risk Streptococci: *S. pneumoniae*, *S. pyogenes* (GAS), *S. dysgalactiae* (GCS), *S. agalactiae* (GBS), *S. viridans group* (*S. salivarius*, *S. anginosus/constellatus*, *S. thermophilus*)
d = High Risk Streptococci: *S. bovis* (*S. gallolyticus, S. infantarius* (GDS)), *S. viridans* group (*S. mitis/cristatus, S. sanguinis, S. gordonii, S. parasanguinis, S. sanguinis, S. mutans*), Nutritionally variant strep (NVS): *Abiotrophia defectiva*, *Granulicatella adiacens*
Abbreviations: +LR = positive likelihood ratio; -LR = negative likelihood ratio; CI = confidence interval, NA = not available, MSSA = methicillin-susceptible *Staphylococcus aureus*, MRSA = methicillin-resistant *Staphylococcus aureus, E. faecalis = Enterococcus faecalis*

© 2025 Freling SR et al. *JAMA Network Open*.
