# Supplementary material for: Interpreting Blood Culture Results as Early Guidance for Infective Endocarditis
Source: JAMA Netw Open. 2025 May 1;8(5):e258079. doi: 10.1001/jamanetworkopen.2025.8079 (PMC12046426; doi:10.1001/jamanetworkopen.2025.8079)
Supplement: Supplement 2. — Data Sharing Statement [file jamanetwopen-e258079-s002.pdf]

Data Sharing Statement

Freling. Interpreting Blood Culture Results as Early Guidance for Infective Endocarditis. *JAMA Netw Open*. Published May 01, 2025. doi:10.1001/jamanetworkopen.2025.8079

Data
Data available: No

Additional Information
Explanation for why data not available: Due to institutional policy, to be HIPAA compliant, we would not like to publish the individual patient data; however, if this is absolutely required then we are open to the conversation of de-identified data.
